# Identification of early indicators of altered metabolism in normal development using a rodent model system

**DOI:** 10.1242/dmm.031815

**Published:** 2018-03-01

**Authors:** Ashok Daniel Prabakaran, Jimsheena Valiyakath Karakkat, Ranjit Vijayan, Jisha Chalissery, Marwa F. Ibrahim, Suneesh Kaimala, Ernest A. Adeghate, Ahmed Hassan Al-Marzouqi, Suraiya Anjum Ansari, Eric Mensah-Brown, Bright Starling Emerald

**Affiliations:** 1 Department of Anatomy, College of Medicine and Health Sciences, United Arab Emirates University, Al Ain, PO Box 17666, Abu Dhabi, UAE; 2 Department of Biology, College of Science, United Arab Emirates University, Al Ain, PO Box 17666, Abu Dhabi, UAE; 3 Department of Biochemistry, College of Medicine and Health Sciences, United Arab Emirates University, Al Ain, PO Box 17666, Abu Dhabi, UAE; 4 Department of Basic Medical Sciences, College of Medicine, Mohammed Bin Rashid University of Medicine and Health Sciences, Dubai Healthcare City, PO Box 505055, Dubai, UAE

**Keywords:** Normal birth weight, Average birth weight, Lower birth weight, Pi3k/Akt and Pparγ signaling, Expression array, DNA methylation, Dnmt1 repressor complex

## Abstract

Although the existence of a close relationship between the early maternal developmental environment, fetal size at birth and the risk of developing disease in adulthood has been suggested, most studies, however, employed experimentally induced intrauterine growth restriction as a model to link this with later adult disease. Because embryonic size variation also occurs under normal growth and differentiation, elucidating the molecular mechanisms underlying these changes and their relevance to later adult disease risk becomes important. The birth weight of rat pups vary according to the uterine horn positions. Using birth weight as a marker, we compared two groups of rat pups – lower birth weight (LBW, 5th to 25th percentile) and average birth weight (ABW, 50th to 75th percentile) – using morphological, biochemical and molecular biology, and genetic techniques. Our results show that insulin metabolism, Pi3k/Akt and Pparγ signaling and the genes regulating growth and metabolism are significantly different in these groups. Methylation at the promoter of the *InsII* (*Ins2*) gene and DNA methyltransferase 1 in LBW pups are both increased. Additionally, the Dnmt1 repressor complex, which includes Hdac1, Rb (Rb1) and E2f1, was also upregulated in LBW pups. We conclude that the Dnmt1 repressor complex, which regulates the restriction point of the cell cycle, retards the rate at which cells traverse the G1 or G0 phase of the cell cycle in LBW pups, thereby slowing down growth. This regulatory mechanism mediated by Dnmt1 might contribute to the production of small-size pups and altered physiology and pathology in adult life.

## Abstract (precis)

**Summary:** This study suggests an important link between the early embryonic environment and later adult physiology and pathology. At least one process by which this might be coordinated is through the regulatory mechanisms mediated by Dnmt1.

## INTRODUCTION

Evidence from a wide variety of molecular, epidemiological and meta-analytical studies has linked the quality of later adult life to the early maternal developmental environment, and showed that variations in them contribute to an increased risk of metabolic diseases, such as type 2 diabetes, obesity, hyperlipidemia, insulin resistance and hypertension in later adult life (Barker and Osmond, 1986; Barker et al., 1989; Hales et al., 1991; Hales and Barker, 1992; Barker, 1995; Park et al., 2008; Godfrey et al., 2011). It has been suggested that smallness at birth is influenced by the developmental environment in that it might be an adaptive mechanism for survival, at the expense of an increased risk for several metabolically associated conditions in later life. Thus a small-size fetus whose development is compromised in the early stages of development because of the mother's nutritional status is more likely to develop a metabolic profile that is appropriate for a low nutrient environment. When, however, the fetus encounters a high-nutrient environment in later life, it would probably adjust in a manner that predisposes it to metabolic diseases, such as type 2 diabetes, obesity, hyperlipidemia and insulin resistance (Gluckman and Hanson, 2004; Simmons, 2005; Barnes and Ozanne, 2011; Gluckman et al., 2011; Vickers, 2014).

Although these observations are significant, most of these data have been obtained from animal studies using experimentally induced intrauterine growth restriction (IUGR) (Vickers et al., 2000, 2003; Simmons et al., 2001; Simmons, 2005; Park et al., 2008). Thus, these results leave open the question as to whether such alterations in processes exist within normal development and birth as well. Finding an answer to this question is important because of the widely accepted viewpoint that the increase in metabolic diseases is not only caused by genetic background but also the early developmental environment. The need to understand the molecular and epigenetic regulatory mechanisms that mediate these processes has become even more important, with the observation that administration of exogenous leptin could reverse changes in Pparα expression (Vickers et al., 2005), and that omega3 fatty acids can reduce hyperleptinemia and hypertension (Wyrwoll et al., 2006).

To address how shuttle changes within the normal, early embryonic developmental period induce alterations in the metabolic profile of the embryo, and to identify the molecular mechanisms mediating these processes, we used birth weight as a marker and established two groups of Wistar rat pups – lower birth weight (LBW) (5th to 25th percentile) and average birth weight (ABW) (50th to 75th percentile) – based on their birth weights. The uterine horns of rodents are vascularized by the uterine and ovarian arteries and, as shown by Even et al. (1994), the level of nutrition of embryos depends on their uterine horn positions, and so birth weights of pups are indicators of the status of *in utero* nourishment. Using these groups, we assessed the basic metabolic parameters, such as pattern of weight increase, changes in blood glucose levels and glucose tolerance, and found them to be different. We also verified the percentage of insulin- and glucagon-secreting β and α cells, status of key insulin signaling pathway molecules, expression of DNA methyltransferases and level of methylation at the *InsII* promoter, and found them to be altered in the metabolically relevant tissues of the pancreas and skeletal muscle (soleus and gastrocnemius).

To establish further how the changes in the basic metabolic parameters alter the growth trajectory of the embryo, and to identify the potential early indicators of this altered metabolic function, we performed a microarray analysis of skeletal muscle from 1-day-old LBW and ABW neonates, because skeletal muscle accounts for the greatest amount of insulin-stimulated glucose disposal in the body (DeFronzo et al., 1985). We observed that several functionally important genes that influence embryonic development are significantly and differentially expressed.

Peroxisome proliferator-activated receptor gamma (Pparγ) signaling plays key roles in energy homeostasis, metabolic diseases and mediates the action of thiazolidinediones, potent insulin sensitizers and highly effective oral medications for type 2 diabetes (Lehmann et al., 1995; Wilcox et al., 2008). Pparγ acts by binding to Ppar-responsive regulatory element (PPRE) as an obligate heterodimer with retinoid X receptors (Rxr proteins) and controls different cellular processes, including regulation of lipid and carbohydrate metabolism (Evans et al., 2004; Barish et al., 2006; Ahmadian et al., 2013). Both Paprγ and Rxrα expression were reduced in LBW pups. In addition, we have demonstrated that adiponectin (AdipoQ), a hormone secreted by adipocytes, which stimulates glucose utilization and fatty-acid oxidation in muscle and has been proposed as a mediator of Pparγ signaling in muscle (Yamauchi et al., 2002; Ahmadian et al., 2013), is a direct transcriptional target of Pparγ and is altered in LBW pups.

One important question that prompted this analysis was how is growth regulated by the early maternal environment and how does that correlate with the birth weight of the fetus? Our results suggest that DNA methyltransferase 1 (Dnmt1) links the maternal environment by increasing methylation in the promoters of genes responsive for embryonic growth, as well as by forming a repressive complex with Hdac1, Rb (Rb1) and E2f1 (Robertson et al., 2000). This repressor complex, we propose, regulates the restriction point and slows down growth by retarding entry from the G1 to S phase of the cell cycle.

## RESULTS

### The birth weights of Wistar rat pups are different at birth

The litter size of rats at birth varies from six to 12 pups. These pups are located in the uterine horns, which are vascularized and nourished by the uterine and ovarian arteries, which anastomose along the length of the uterine horns. We opined that if the developmental environment is so closely integrated with the growth of embryos, then it should be possible to detect changes in their sizes, depending on their location in the uterine horns. To verify this, we mated female rats of similar weight (200-210 g) and age with males of closely matched weights (340-350 g) and age. This non-sister-brother mating procedure was repeated for three generations to keep the variation in weight of the animals used for the study to the minimum. To confirm the apparent variation in weights, if any, we assessed the birth weights of pups born to third generation females (parental generation, P) and the weights varied between 4.6 and 7.2 g. Based on the observation of ∼250 pups, we grouped them into percentiles by their weights as follows: >4.94 g, 5th; >5.5 g, 25th; >6.2 g, 50th; >6.95 g, 75th; and >7 g, 90th percentile. To minimize variation, we picked pups between the 50th and 75th percentiles born to different parents (F1), and set up crosses, when the weights of the female animals were ∼200-210 g, with males of weight 340-350 g. This was repeated and the F2 generation was grouped at birth into two groups; namely, LBW (5th to 25th percentile; 5-5.4 g) and ABW (50th to 75th percentile; 6.2-6.9 g). This was done to ensure that we could classify the pups into distinct physiological groups so that any metabolic, developmental, genetic and epigenetic differences that might occur would be distinct, as opposed to the gradual and minimal changes that would occur if we employed pups with only minimal variations in the birth weight.

### ABW and LBW pups have different metabolic parameters

**Fig. 1.**
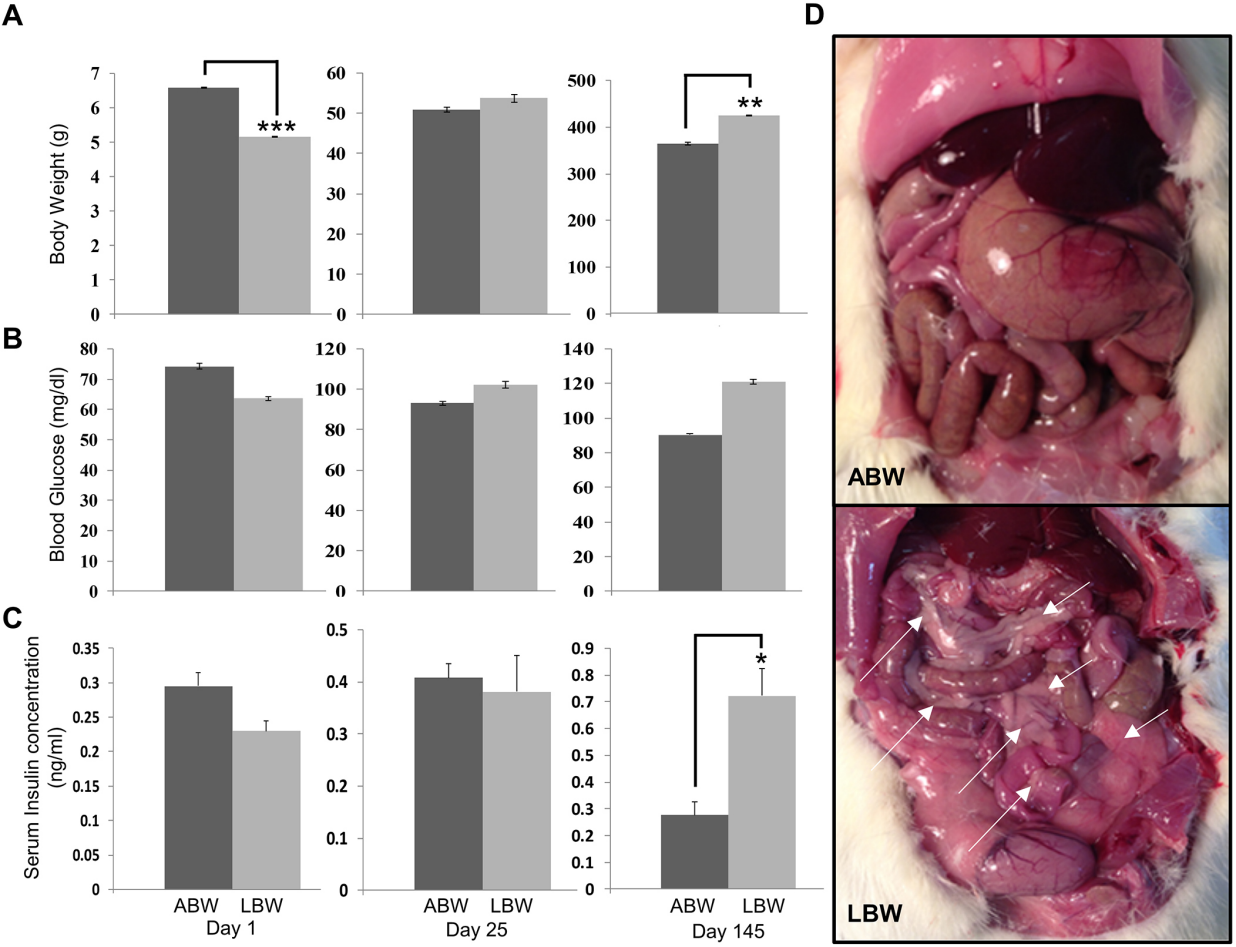
**Growth trajectory of rat pups is influenced by birth weight.** (A) The body weight of ABW and LBW pups at birth (day 1), after weaning (day 25) and at adulthood (day 145). (B) Glycemia of ABW and LBW pups at birth (day 1), after weaning (day 25) and at adulthood (day 145). (C) Serum insulin levels of ABW and LBW pups at birth (day 1), after weaning (day 25) and at adulthood (day 145). (D) The presence of visceral fat in adults of LBW pups (arrows) and their absence in ABW adults. *P<0.05, **P<0.01, ***P<0.001, two-tailed unpaired Student's *t*-test.

Using pups of the two distinct birth weight groups, we analyzed basic metabolic parameters such as the pattern of weight increase, changes in blood glucose at birth (day 1), at weaning (day 25) and at adulthood (day 145) to determine whether there were any differences and if so, whether these differences lead to changes in their growth trajectories. The birth weights of LBW pups were ∼22% less than those of the ABW pups at birth (5.15±0.022 g versus 6.58±0.019 g; *P*<0.001), but by day 25, the weights of the LBW pups had increased by 110% compared to those of the ABW pups (53.67±0.05 g versus 50.79±0.88 g). At day 145 (adult), the LBW pups were significantly heavier (424.4±1.35 g versus 365.06±1.63 g; Fig. 1A) (*P*<0.01), with an increase of ∼117%, compared to the ABWs (Fig. 1A).

In comparison to those of ABW pups, blood glucose levels were lower in the LBW pups at day 1 (74.27±0.65 mg/dl versus 63.62±1.01 mg/dl). The blood glucose levels were approximately equal between the groups at the time of weaning (102.2±1.73 mg/dl for LBW and 93±1.06 mg/dl for ABW), but by day 145, the LBW pups were hyperglycemic while the ABWs remained normoglycemic (121±1.35 mg/dl and 90.25±1.63 mg/dl) (Fig. 1B). Although the fasting serum insulin levels did not show any significant differences between ABW and LBW pups at day 1 and at weaning (day 25), there was a significant increase in the fasting serum insulin levels of 145-day-old LBW adult animals, indicating insulin resistance, which also supports the increased fasting glucose levels observed (Fig. 1C). Thus, there seems to be evidence of insulin resistance in adult LBW animals, which also had high fasting glucose levels (Fig. 1B). In addition, examination of the internal organs of adults from the two groups revealed the presence of large amounts of visceral fat in adults from LBW pups, which was not discernible around the internal organs of ABW adults (Fig. 1D). These results are suggestive of the fact that the early developmental environment is important for normal growth and metabolism of the embryo, and that changes in the maternal environment within the normal birth range could lead to changes in growth trajectories.

### Percentage of insulin-secreting β cells and glucagon-secreting α cells and their expression differ between ABW and LBW pups

**Fig. 2.**
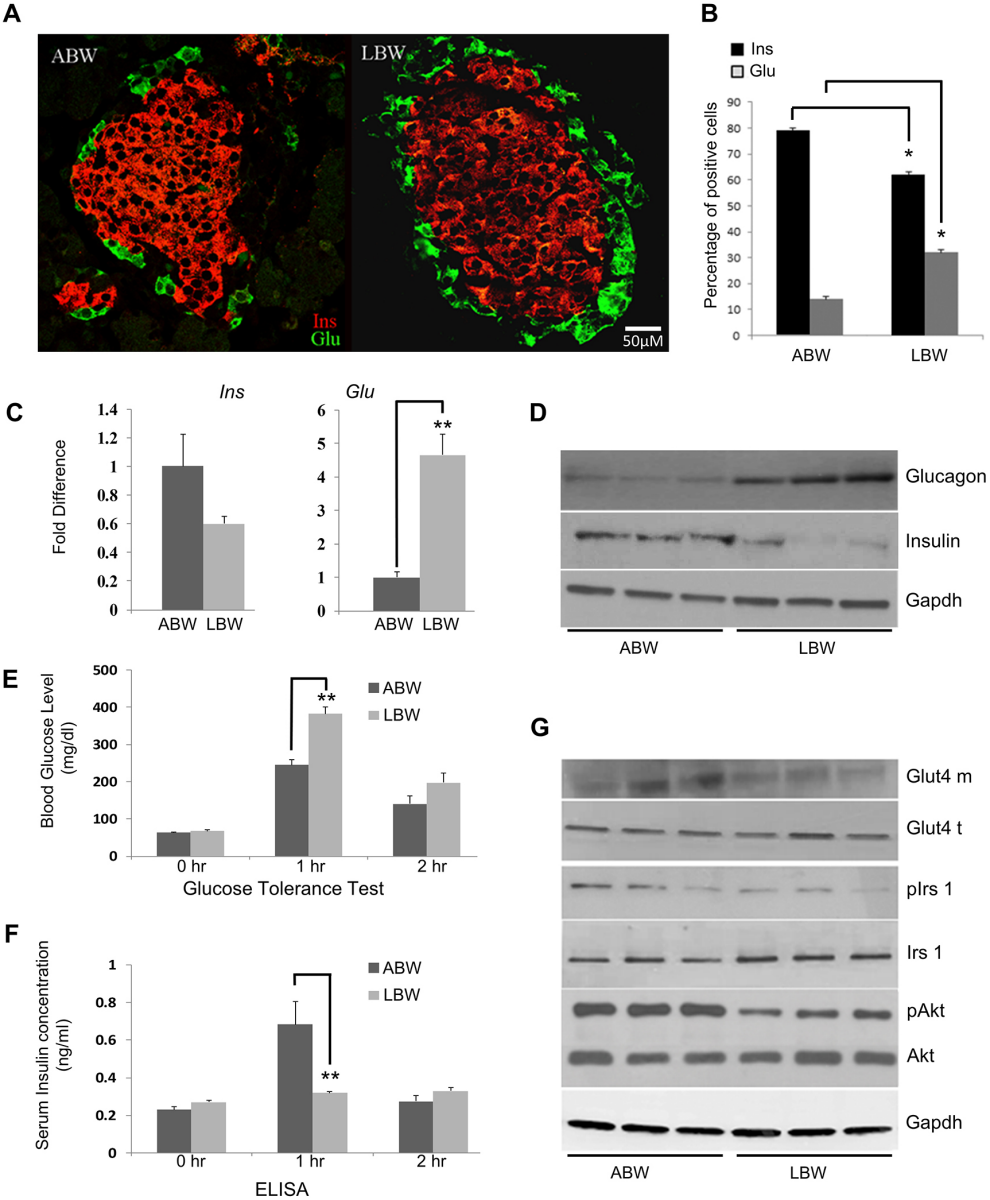
**Expression of insulin, glucagon and the Pi3k/Akt pathway genes differs between ABW and LBW pups.** (A) Distribution of insulin-secreting β cells (green) and glucagon-secreting α cells (red) in the islets of the pancreas of 1-day-old ABW and LBW pups (scale bar: 50 µm). (B) Percentage of insulin-secreting β cells (green) and glucagon-secreting α cells (red) in 1-day-old ABW and LBW pups. (C) qRT-PCR showing fold changes in expression of mRNAs of insulin and glucagon in pancreata of 1-day-old ABW and LBW pups. 18S was used to normalize the expression. (D) Differences in the level of insulin and glucagon expression, determined by western blot analysis, between 1-day-old ABW and LBW pups. Gapdh was used as a loading control. (E) Glucose tolerance test at 0, 1 h and 2 h after 6 h fasting and administration of 2 mg/kg body weight of glucose in 1-day-old ABW and LBW pups. (F) Serum insulin concentration at 0, 1 h and 2 h after 6 h fasting and administration of 2 mg/kg body weight of glucose determined by ELISA in 1-day-old ABW and LBW pups. (G) Western blot analysis of the components of the Pi3k/Akt pathway (Akt, Irs1, Glut4) showing a reduction in the phosphorylated levels of Akt and Irs1, and the membrane fraction of Glut 4 in LBW pups. Gapdh was used as a loading control. *P<0.05, **P<0.01, two-tailed unpaired Student's *t*-test.

Having observed changes in the glucose levels and growth rates of the different groups of pups, we next examined the expression of insulin and glucagon in the pancreatic islets of ABW and LBW pups by immunohistochemistry, to determine whether any differences existed between the two groups of pups. In general appearance, there was a clear increase in the expression of glucagon in LBW pups, but the difference in expression of insulin between the two groups was not so obvious. In several of the sections of LBW islets, large areas of glucagon-containing cells completely surrounded the centrally located insulin-containing cells (Fig. 2A).

To record the changes more accurately, we determined the percentage of insulin- and glucagon-positive cells in pancreata of these pups. As shown in Fig. 2B, the mean percentage of insulin-containing cells in the ABW group was significantly higher than that in the LBW group (75.14±6.8% versus 63.8±5.21%; *P*<0.05) but the mean percentage of glucagon cells was significantly higher in the LBW group (10.01±1.02% versus 34.42±2.5%; *P*<0.05). These semi-quantitative determinations were further confirmed by real time RT-PCR and western blot analyses. The results of both analyses showed a significantly higher expression of both glucagon mRNA and protein in islets of LBW pups compared to that in the ABW pups, while the expression of insulin was lower in LBW pups (Fig. 2C,D).

### The Pi3k/Akt signaling pathway is altered in LBW pups

Although there was a clear difference in the levels of the expression of insulin and glucagon, as well as the percentages of α and β cells in pancreatic islets, there was no significant difference in the fasting blood glucose levels of the 1-day-old pups. After 6 h of fasting, a glucose tolerance test (GTT) was performed on the two groups of pups to confirm their glycemic-regulating capabilities. Although blood glucose levels returned to normal levels in both groups after 2 h, the glucose levels at 1 h were significantly higher in LBW pups than in the ABW pups (*P*<0.01) (Fig. 2E). We also tested the serum insulin levels after 6 h of fasting, during the GTT, on the two groups of pups using enzyme-linked immunosorbent assay (ELISA). As shown in Fig. 2F, there were significantly higher levels of serum insulin in ABW pups than in LBW pups after 1 h (Fig. 2F). In an effort to further explore the consequences of the differences in birth weight and in the expression of pancreatic insulin and glucagon of ABW and LBW pups, we analyzed the expression of the members of the Pi3k/Akt signaling cascade by western blotting, using skeletal muscle tissue from these pups. The Pi3k/Akt signaling pathway regulates both glycolysis and the enzymes that regulate the pathway itself. As shown in Fig. 2G, while there was no difference in the total expression of Akt and Irs1 in ABW and LBW pups, their phosphorylated moieties were downregulated in LBW pups. Similarly, while the overall expression of the glucose transporter Glut4 was not altered in the two groups, its membrane fraction was reduced in LBW pups (Fig. 2G), suggesting that not only was there a difference in the growth and development of the two groups but the signaling pathways that regulated metabolism in ABW and LBW pups also differed.

### Microarray analyses identify the differentially expressed genes between LBW and ABW pups

**Fig. 3.**
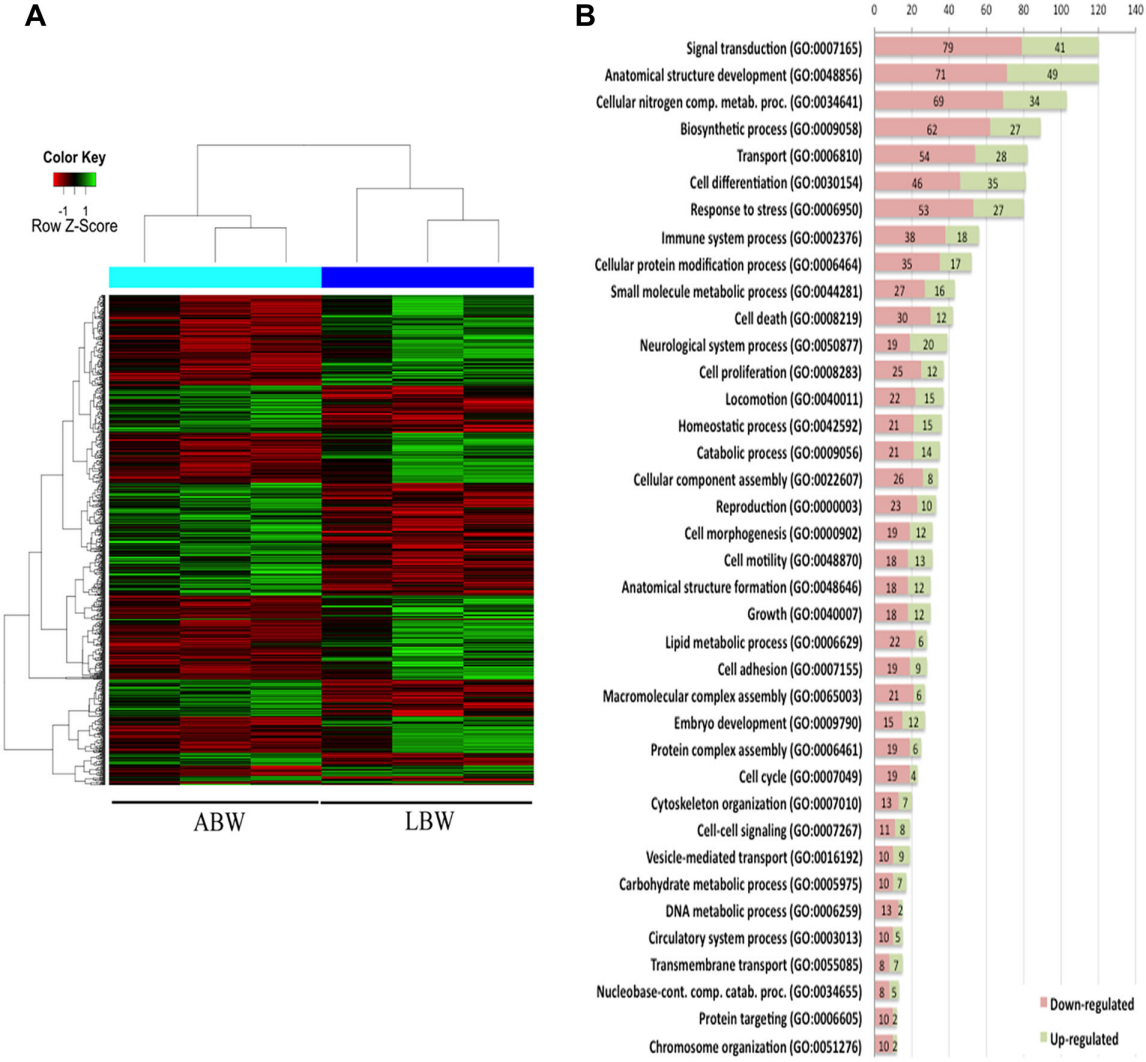
**Expression of genes that regulate growth and development vary between ABW and LBW pups.** (A) Heat maps from microarray analyses of skeletal muscles of ABW and LBW showing genes that are altered in ABW and LBW pups. (B) GO terms and number of genes and their pathways are affected differently between ABW and LBW pups.

To gain an insight into changes in the genes and the pathways they regulate that might be contributing to changes in metabolism, development and growth, we carried out whole-genome transcriptional profiling analysis of skeletal muscle from ABW and LBW pups. Our results revealed a total of 1047 genes that were significantly and differentially expressed between LBW and ABW pups (*P*<0.05). Of these, 577 genes were upregulated and 470 genes were downregulated in LBW samples (Fig. 3A). Tables S1 and S2 list the top 40 genes for which expression was either downregulated or upregulated, respectively, in LBW pups (a complete list is provided in Tables S3 and S4).

The significantly and differentially expressed genes were grouped according to their functional significance using Gene Ontology (GO) terms. The genes enriched for each GO term were further classified into the number of genes upregulated or downregulated by at least 1.5 fold. These genes were classified into 38 functional categories; namely, signal transduction (79 downregulated and 41 upregulated genes), anatomical structure development (71 downregulated and 49 upregulated genes), cellular nitrogen compound metabolic process (69 downregulated and 34 upregulated genes), biosynthetic process (62 downregulated and 27 upregulated genes), transport (54 downregulated and 28 upregulated genes), cell differentiation (46 downregulated and 35 upregulated genes), response to stress (53 downregulated and 27 upregulated genes), immune system process (38 downregulated and 18 upregulated genes), cellular protein modification process (35 downregulated and 17 upregulated genes), small molecule metabolic process (27 downregulated and 16 upregulated genes), cell death (30 downregulated and 12 upregulated genes), neurological system processes (19 downregulated and 20 upregulated genes), cell proliferation (25 downregulated and 12 upregulated genes), locomotion (22 downregulated and 15 upregulated genes), homeostatic process (21 downregulated and 15 upregulated genes), macromolecular complex assembly (21 downregulated and 6 unregulated genes), catabolic process (21 downregulated and 14 upregulated genes), cellular component assembly (26 downregulated and 8 upregulated genes), reproduction (23 downregulated and 10 upregulated genes), cell morphogenesis (19 downregulated and 12 upregulated genes), cell mobility (18 downregulated and 13 upregulated genes), anatomical structure formation (18 downregulated and 12 upregulated genes), growth (18 downregulated and 12 upregulated genes), lipid metabolic processes (22 downregulated and 6 upregulated genes), cell adhesion (19 downregulated and 9 upregulated genes), embryo development (15 downregulated and 12 upregulated genes), protein complex assembly (19 downregulated and 6 upregulated genes), cell cycle (19 downregulated and 4 upregulated genes), cytoskeleton organization (13 downregulated and 7 upregulated genes), cell-cell signaling (11 downregulated and 8 upregulated genes), vesicle-mediated transport (10 downregulated and 9 upregulated genes), carbohydrate metabolic process (10 downregulated and 7 upregulated genes), DNA metabolic process (13 downregulated and 2 upregulated genes), circulatory system process (10 downregulated and 5 upregulated genes), transmembrane transport (8 downregulated and 7 upregulated genes), nucleobase-cont. comp. catabolic processes (8 downregulated and 5 upregulated genes), protein targeting (10 downregulated and 2 upregulated genes) and chromosome organization (10 downregulated and 2 upregulated genes) (Fig. 3B).

### Quantitative real-time PCR validates gene expression array results

In order to validate the microarray expression results, we performed qRT-PCR experiments on a selection of downregulated and upregulated genes from the microarray using the same RNA samples from skeletal muscles used for the microarray analyses. Seven downregulated genes; namely, aminoadipate aminotransferase (*Aadat*) (NM_017193), potassium channel tetramerization domain-containing 21 (*Kctd21*) (XM_001064087), DnaJ (Hsp40) homolog, subfamily C, member 28 (*Dnajc28*) (NM_001014124), endothelin 1 (*Edn1*) (NM_012548), leptin (*Lep*) (NM_013076), patched domain containing 1 (*Ptchd1*) (XM_001056385), peroxisome proliferator-activated receptor gamma (*Pparg*) (NM_013124); and six upregulated genes, matrix metallopeptidase 13 (*Mmp13*) (XM_001072242), secreted phosphoprotein 1 (*Spp1*) (NM_012881), integrin-binding sialoprotein (*Ibsp*) (NM_012587), bone gamma-carboxyglutamate protein (*Bglap*) (NM_013414), matrix metallopeptidase 8 (*Mmp8*) (NM_022221) and matrix metallopeptidase 9 (*Mmp9*) (NM_031055), were analyzed. 18S RNA expression was used as a normalization standard. qRT-PCR results confirmed that the changes in expression were comparable to the values obtained from the microarray (Tables S1 and S2), suggesting that the genes we had identified from the array were genuine targets for which expression had been altered in LBW pups.

### The expression of Pparγ, which regulates fatty acid storage and glucose metabolism, is reduced in LBW pups

**Fig. 4.**
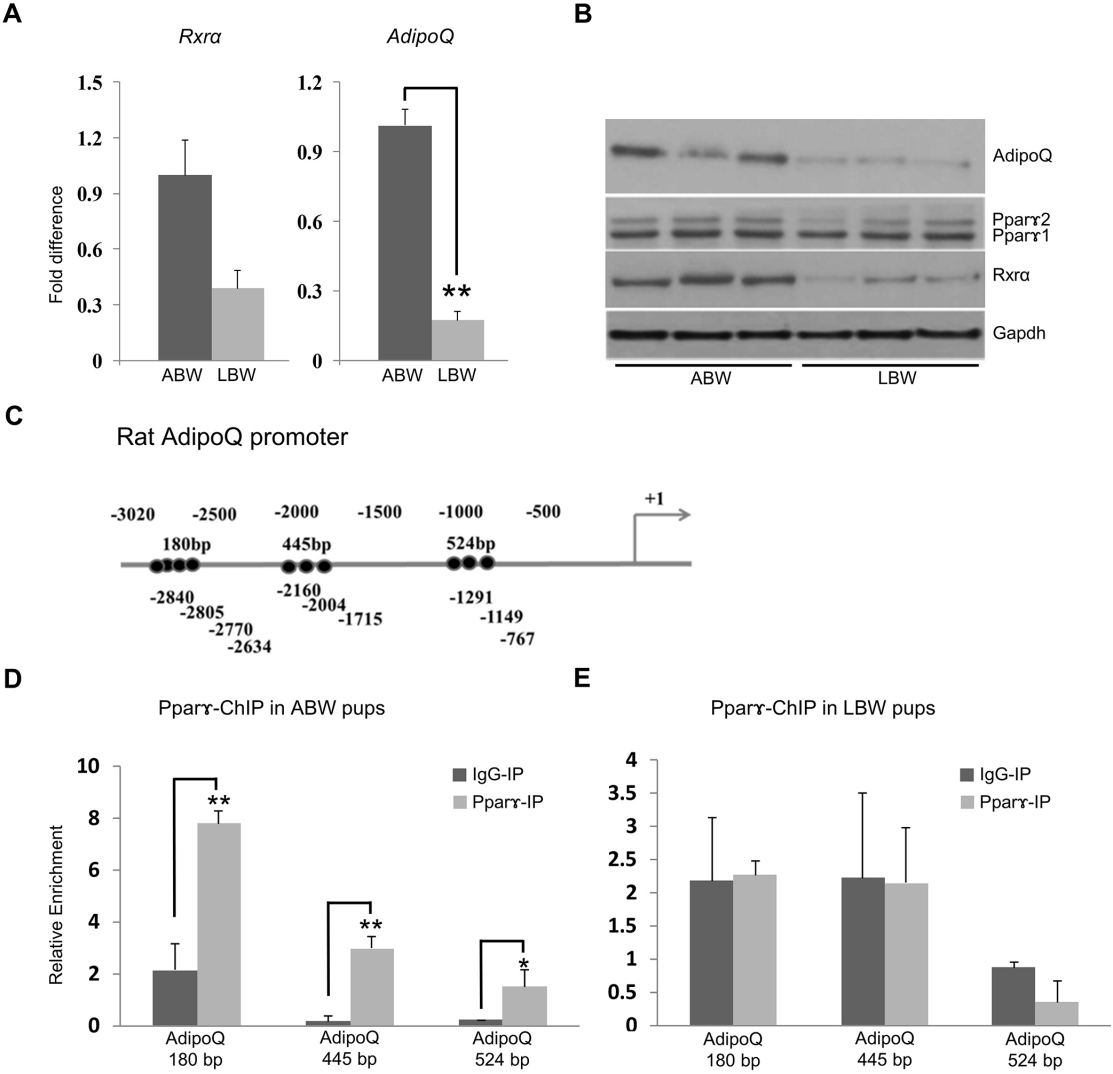
**Expression of the *Pparg* gene and its targets are different in ABW and LBW pups.** (A) qRT-PCR reveals a reduction in the expression of *Rxra* and *AdipoQ* in LBW compared to ABW pups at day 1. (B) Western blot analysis showing the reduction in the expression of Rxrα, Pparγ 1 and 2 and AdipoQ in LBW pups. (C) The rat adiponectin gene promoter showing the position of PPRE elements tested in this study (+1 is the transcription start site). (D,E) ChIP analysis of the occupancy of Pparγ in the adiponectin promoter of ABW (D) and LBW (E) pups. *P<0.05, **P<0.01, two-tailed unpaired Student's *t*-test.

One of the genes for which expression was downregulated in LBW pups was *Pparg*, a member of a nuclear receptor superfamily of ligand-inducible transcription factors that plays key roles in energy homeostasis and metabolic diseases (Tontonoz and Spiegelman, 2008). We decided therefore to analyze further, by western blotting and qRT-PCR, the expression of Pparγ in ABW and LBW pups. It is noteworthy that while both the mRNA and protein expression of Pparγ was reduced in LBW skeletal muscle, western blotting showed that the decrease was mainly due to Pparγ2 protein (Fig. 4B; Table S1). As Pparγ always binds to its targets as heterodimers with Rxrs, we also examined the expression of Rxrα and, not surprisingly, Rxrα expression was reduced in LBW skeletal muscle in comparison to that in ABW pups (Fig. 4A,B).

Pparγ also positively regulates glucose uptake in insulin-sensitive tissues, including skeletal muscles, by targeting adiponectin (AdipoQ). Because both Pparγ and Rxrα expression were reduced and insulin signaling was altered in LBW pups, we checked whether the expression of AdipoQ was also altered in LBW skeletal muscle. As was observed with Pparγ and Rxrα expression, the expression of AdipoQ was reduced in LBW skeletal muscle (Fig. 4A,B), suggesting that at least one pathway that is altered in LBW pups as a result of differences in the early maternal environment is Pparγ signaling.

It could be argued that the reduction in the expression of AdipoQ was a consequence of changes in the overall signaling, rather than a change in the direct transcriptional regulation by Pparγ. To verify this possibility, we used the *in slico* program PPRESearch to identify any possible peroxisome proliferator response elements (PPREs) in the promoter region of the rat *AdipoQ* gene (Venkatachalam et al., 2009). In the promoter region, between −3010 to −500 bp upstream of the transcription start site, we identified three probable regions with multiple PPREs: a 180 bp region with four probable PPREs at −2840 to −2634, a 445 bp region with three PPREs at −2160 to −1715 and a 524 bp region with three PPREs at −1291 to −767 (Fig. 4C). We verified the binding of Pparγ to these PPREs in ABW and LBW skeletal muscle using chromatin immunoprecipitation (ChIP) analyses, and observed significantly lower binding in all the three regions in LBW tissues (Fig. 4D,E). These results suggest that the identified PPREs at the *AdipoQ* promoter region are functional binding sites of Pparγ, and the changes in the expression of AdipoQ resulted from changes in the transcriptional regulation of the *AdipoQ* promoter by Pparγ.

### DNA methyltransferases are expressed differently in ABW and LBW rat pancreas at day 1

**Fig. 5.**
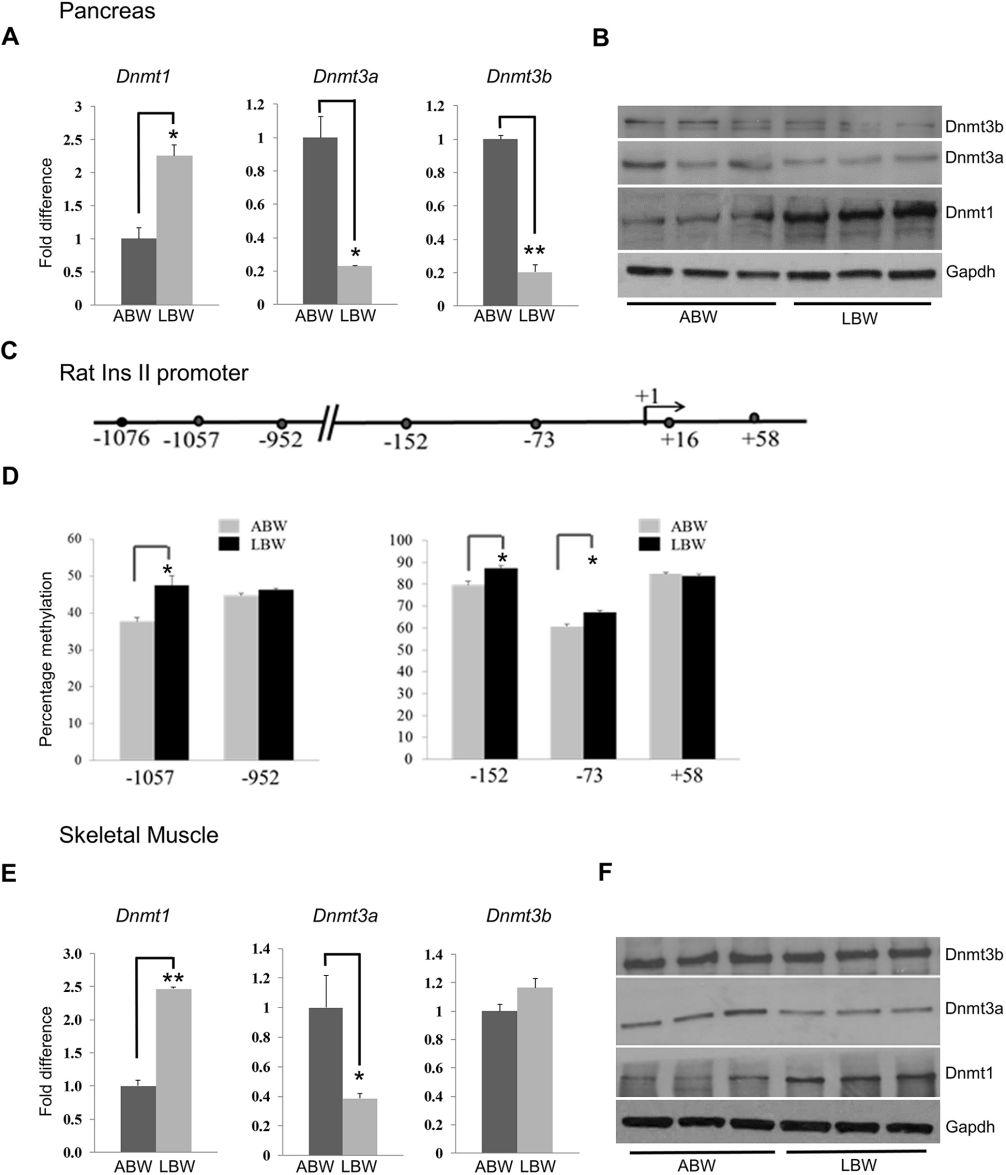
**Expression of DNA methyltransferases in the pancreas and skeletal muscle, and methylation of the *InsII* promoter in pancreata, differ between ABW and LBW pups.** (A) qRT-PCR analysis of the expression of DNA methyltransferases *Dnmt1*, *Dmnt3a* and *Dnmt3b* in the pancreas of ABW and LBW pups at day 1. (B) Western blot analysis of the expression of DNA methyltransferases reveal an increase in the expression of Dnmt1, but a decrease in Dnmt3a and Dnmt3b expression, in the pancreas of LBW pups. (C) The rat *InsII* promoter depicting the position of CPGs analyzed with the Sequenom mass array system. (D) Percentage methylation of CpGs analyzed by the Sequenom microarray system in ABW and LBW pups. (E) qRT-PCR analyses showing differences in the expression of DNA methyltransferases *Dnmt1*, *Dnmt3a* and *Dnmt3b* in skeletal muscle of ABW and LBW pups. Expression of *Dnmt1* is increased, expression of *Dnmt3a* is decreased, and the expression of *Dnmt3b* remains unaltered in the two groups. (F) Western blot analyses of the expression of DNA methyltransferases Dnmt1, Dnmt3a and Dnmt3b reveal an increase in Dnmt1 but a decrease in Dnmt3a, without any significant change in the expression of Dnmt3b, in LBW pups. *P<0.05, **P<0.01, two-tailed unpaired Student's *t*-test.

The differences in the expression of genes on a global level, which appear to be well coordinated, prompted us to analyze the role of epigenetic mechanisms in the regulation of these processes. DNA methylation is an important epigenetic regulatory mechanism in living organisms and it is regulated by three methyltransferases: Dnmt1, DNA methyltransferase 3a (Dnmt3a) and DNA methyltransferase 3b (Dnmt3b) (Bird, 2002). To verify the probable role of DNA methylation, we analyzed the expression of the three DNA methyltransferases by qRT-PCR and western blotting. While the expression of Dnmt1 was increased, the expression of Dnmt3a and Dnmt3b was decreased significantly in LBW pancreata (Fig. 5A,B). These differences in Dnmt protein expression suggest that DNA methylation mediated by Dnmt proteins might be playing a role in the altered metabolic profiles of ABW and LBW pups, and could be involved in the coordination of growth on a global scale.

### Methylation of the *InsII* promoter differs between ABW and LBW rat pancreas

The differences in the expression of Dnmt proteins, especially the increased expression of Dnmt1, which is responsible for maintaining methylation during the early stages of development, and the decrease in the expression of insulin in LBW pups at day 1 prompted us to verify the level of DNA methylation at the promoter of the *InsII* gene. We employed the Sequenom Massarray system to analyze the percentage methylation at the promoter of the *InsII* gene using genomic DNA isolated from β cells of 1-day-old ABW and LBW pups (Lacy and Kostianovsky, 1967; Lernmark, 1974). We used two sets of primers covering the promoter regions −1136 to −835 and −245 to +118 (Fig. 5C). Of the seven CpGs present in these regions, we could assess the percentage methylation of five of them. Of these five, three CpGs (−1057, −152 and −53) showed significant increases in the percentage of methylation in pancreatic β cells from LBW pups (Fig. 5D).

### Regulation of the cell cycle is different in ABW and LBW pups

One of the important observations which prompted this investigation was the variation in the sizes of pups at birth. The increase in the expression of Dnmt1 in the pancreas of LBW pups, along with changes observed in DNA methylation, appeared to implicate Dnmt1 as an important link between size of pups and the early embryonic developmental environment. This is based on the fact that Dnmt1, in complex with Hdac1, Rb and E2f1, has been shown to act as a repressor complex that controls the restriction check point, a slowing down mechanism of the cell cycle and thereby of growth. As a first step to verify a possible role of this Dnmt1 repressor complex, we analyzed the expression of the three Dnmts by qRT-PCR and western blotting using skeletal muscle from ABW and LBW pups. The expression of Dnmt1 was significantly increased in LBW skeletal muscles, but, unlike in the pancreas, where the expression of both Dnmt3a and Dnmt3b were decreased, only the expression of Dnmt3a decreased significantly in LBW skeletal muscle (Fig. 5E,F).

**Fig. 6.**
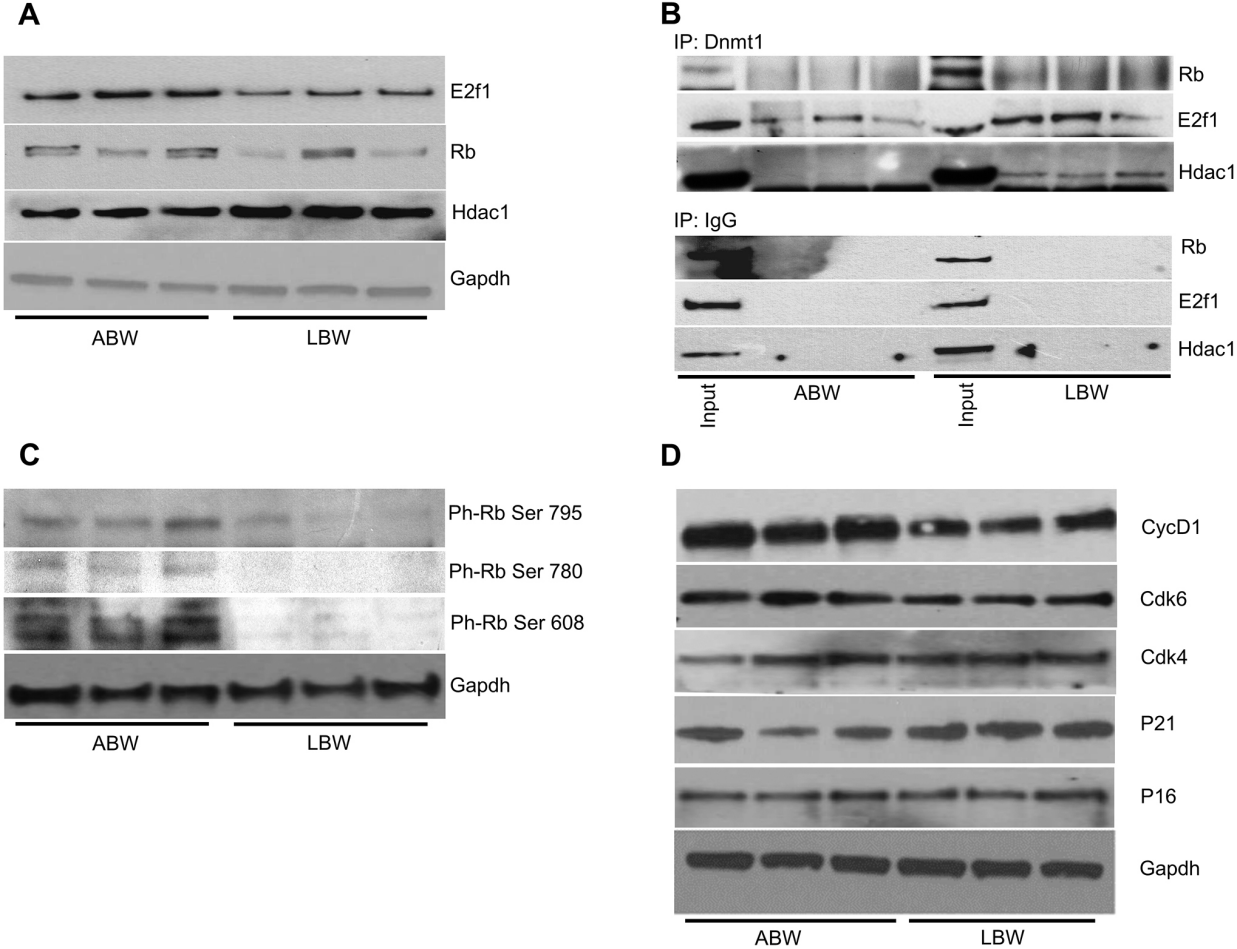
**Regulation of the cell cycle is different in ABW and LBW pups.** (A) Western blot analyses of the skeletal muscle of members of the Dnmt1 repressor complex, E2f1, Rb and Hdac1, in ABW and LBW pups at day 1. Gapdh was used as a loading control. (B) Co-IP analysis of the Dnmt1 repressor complex using anti-Dnmt1 antibody, and western blot analysis of the members of the complex E2f1, Rb, Hdac1, in ABW and LBW pups at day 1. Co-IP using IgG was used as a control; 5% of the total extract was used as input. (C) Western blot analysis of skeletal muscle of 1-day-old ABW and LBW pups for phospho Rb (ser 608, ser 780 and ser 795). Gapdh was used as a loading control. (D) Western blot analysis of cyclin D1 and cyclin-dependent kinases Cdk4 and Cdk6, and the G1 phase cell cycle inhibitors P16 and P21, using skeletal muscle of ABW and LBW pups at day 1. Gapdh was used as a loading control.

Having confirmed the change in the expression of Dnmt1 in skeletal muscle, we next assessed whether there was any change in the expression of the members of the Dnmt1 repressor complex, Hdac1, Rb and E2f1, by western blotting. The expression of Rb and E2f1 was reduced in LBW pups, while there was a slight increase in the expression of Hdac1 (Fig. 6A). It is of interest to point out that an increase in Hdac1 expression was shown to inhibit Pparγ expression in adipose tissue (Fu et al., 2005).

To further verify the role of this complex in coordinating growth, we performed a co-immunoprecipitation (Co-IP) experiment using an antibody to Dnmt1, and analyzed the expression of the members of the Dnmt1 repressor complex, Hdac1, Rb and E2f1. The expression of all three members was increased in LBW skeletal muscles (Fig. 6B). IgG was used as control in all Co-IP experiments. The fact that the expression of the Dnmt1 repressor complex was increased in LBW pups, which in turn restricts the level of E2f1 available for progression of the cell cycle, suggests that at least one mechanism by which growth is altered in LBW pups is the slowing down of progression through the restriction point of the cell cycle by the Dnmt1 repressor complex.

Phosphorylation of Rb is necessary for abrogation of the effect of the repressor complex at the restriction point of the cell cycle. If, however, there is a high level of Rb in the Dnmt1 repressive complex of LBW pups, then the phosphorylation level of Rb could also be low in LBW pups. We verified this possibility by assessing Rb phosphorylation at ser 608, which blocks the E2f1 transactivation domain binding, and ser 780 and ser 795, which were shown to be phosphorylation targets of cyclin D1 and Cdk4/6. Using skeletal muscle from LBW pups, we demonstrated that the phosphorylation levels of Rb at these serines are indeed low in LBW pups (Fig. 6C). These low levels of phospho-Rb (ph-Rb), together with high levels of Rb and E2f1in the repressor complex, imply that there is a low level of E2f1 available for transcription initiation. This might retard the rate of the cell cycle and could be responsible for the smaller size of LBW pups.

Because our results appeared to suggest that the Dnmt 1 repressor complex could be responsible for the availability of E2f1, leading to retardation of cell cycle progression, we analyzed the expression of the early G1-specific cyclins and cyclin-dependent kinases – cyclin D1, Cdk4 and Cdk6 – as well as the cell cycle inhibitors P16 and P21, by western blotting, to determine whether there are any alterations.

The expression of the cyclin-dependent kinases Cdk4 and Cdk6, which are responsible for the early G1 phase of the cell cycle, was not altered in ABW and LBW pups, but the expression of the early G1 cell cycle cyclin, cyclin D1, was decreased in LBW pups. Similarly, the expression of P21, the promoter of the nuclear import of Cdk4/6-cyclin D1 was increased in LBW pups (Fig. 6D), whereas that of the inhibitor, P16, was not altered. P21 binds to Cdk4/6-cyclin D1 and the complex is translocated into the nucleus where it induces phosphorylation of Rb. Phosphorylation of Rb leads to the dissociation of E2f1, which then activates the transcription of target genes. Phosphorylation of Rb, or lack of it, and its effect on E2f1 therefore determine the rate at which cells traverse the G1 or G0 phase of the cell cycle.

We opined that a decrease in the expression of cyclin D1 should be associated with a concomitant decrease in the formation of Cdk4/6-cyclin D1 complex, and therefore less nuclear import, which could be responsible for the observed accumulation of P21 in LBW pups.

## DISCUSSION

It is well established that metabolic diseases, such as type 2 diabetes, obesity and hypertension, are increasing at an alarming rate all over the world. Although overnutrition and the sedentary lifestyle of modernity are recognized as significant players in this modern scourge, it is now becoming increasingly apparent that of even greater significance is the role played by the early embryonic environment. It was Barker (Hales et al., 1991; Barker et al., 1993; Barker, 1995) who first proposed the developmental origins of adult disease based on the observations that birth weight could be inversely correlated with cardiovascular risk. He argued that an optimal fetal developmental environment is crucial for a healthy adult life and that any perturbations during this period will lead to a higher risk of developing metabolic diseases. This relationship between birth weight and the risk of developing metabolic diseases has been supported by a variety of epidemiological studies (Harder et al., 2007). It has also been suggested that the molecular mechanisms which mediate these changes operate within the normal range of birth weights, and involve mechanisms of developmental plasticity rather than pathology (Gluckman et al., 2007, 2008; Emerald et al., 2011). Most studies that have addressed this relationship using animal models of intrauterine growth retardation, either by maternal undernutrition or uterine artery ligation during pregnancy, have shown that offspring born small for their gestational ages are susceptible to different metabolic diseases associated with the sedentary lifestyle, such as obesity, hypertension, hyperinsulinemia and hyperleptinemia, in adulthood (Unterman et al., 1990; Vickers et al., 2000, 2001, 2003; Simmons et al., 2001; Simmons, 2007; Park et al., 2008). If it is true that mechanisms mediating these changes operate within the normal range of birth weights as well, then it should be possible to identify these mechanisms using a rodent model system because they are multiparous, and the birth weights of rodent pups have been correlated with the positions they occupy on the uterine horn during embryonic development (Even et al., 1994). To eliminate the major reasons responsible for the inconsistencies of previous studies highlighted by Morris and Chen (2009), we employed 1-day-old male fetuses bred under similar environmental circumstances and grouped the pups at birth as LBW (5th to 25th percentile) and ABW (50th to 75th percentile) to split them into two distinct physiological groups. Interestingly, the variations in birth weight in the present study were similar to those observed in studies that had employed the experimental manipulations of inducing undernutrition by the obliteration of the uterine artery of the uterine horn during the later stages of pregnancy, although these studies used Spraque-Dawley rats (5.15±0.022 g and 6.58±0.019 g versus 5.96±0.68 and 7.00±0.89 g) (Simmons et al., 2001). If the hypothesis that the maternal nutritional environment is closely linked to the growth of the developing embryo is correct, then undernutrition of mothers during pregnancy should result in further decrease in birth weight. It is noteworthy that in comparison to normally fed mothers, maternal undernutrition during pregnancy led to the birth of small for size pups, and these were indeed smaller than LBW pups, although all the pups born to these mothers were included for the weight assessment (5.15±0.022 and 6.58±0.019 g versus 4.12±0.1 g and 6.8±0.1 g) (Vickers et al., 2008). These results suggest that the molecular mechanisms which mediate these changes operate within the normal range of birth weights and depend on the maternal developmental environment, which could be further aggravated by nutritional variations.

We also have examined the morphological, biochemical, molecular and epigenetic characteristics of the pancreas, the source of insulin and skeletal muscle, which accounts for the majority of insulin-stimulated glucose disposal (DeFronzo et al., 1985) from neonates of ABW and LBW to decipher how and why the variation in weight occurs. Our results show that differences in metabolic parameters, growth trajectories and signaling pathways, although small, do exist between ABW and LBW pups at birth and these may be indicators of later disease risk.

The Pi3k/Akt signaling pathway, an important member of the complex network mediating insulin signaling, is altered in low birth weight humans (Ozanne et al., 2005; Taniguchi et al., 2006; Jensen et al., 2008). In this pathway, Pi3k, a lipid kinase that acts as a heterodimeric enzyme, phosphorylates the D-3 position of the inositol ring in phosphoinositides (Whitman et al., 1988; Carpenter et al., 1990; Stephens et al., 1991) and when insulin secretion is stimulated by the presence of glucose in the circulation, this signaling cascade that phosphorylates the serine-threonine kinase, Akt, is activated. Akt phosphorylation then triggers the uptake and metabolism of glucose through the translocation of the glucose transporters, Glut2 and Glut4, to the plasma membrane (Kan et al., 1994; Hill et al., 1999). Significantly, our study clearly shows that the phosphorylation of Akt, Irs and Glut4 translocation to the membrane are all downregulated in LBW pups, although the signaling pathway is functional.

DNA methylation occurs mainly by the addition of a methyl (CH_3_) group to the fifth carbon molecule of cytosine resulting in the formation of 5-methylcytosine (5-mC), which is required for normal development and differentiation. Alterations in 5-mC have been associated with several disease conditions (Robertson, 2005). Analysis of DNA methylation of the human genome has revealed the existence of widespread differences between different tissues (Lister et al., 2009). In general, 5-mCs are present as CpGs and three Dnmts are responsible for maintaining (Dnmt1) and *de novo* (Dnmt3a and Dnmt3b) DNA methylation (Smith and Meissner, 2013). Expression of insulin, which is crucial for metabolic regulation, has been shown to be regulated by DNA methylation in humans and mice (Kuroda et al., 2009). It has also been shown that the insulin promoter is heavily methylated in most of the tissues studied and is demethylated specifically in pancreatic β-cells (Kuroda et al., 2009). DNA methylation suppresses gene transcription, and because the expression of insulin is downregulated in LBW pups with an increase in the methylation of specific CpGs in the *InsII* promoter, we suggest that DNA methylation, especially by Dnmt1, might play pivotal roles in the changes observed between ABW and LBW pups.

Our analyses suggest that small changes in birth weight could be an indicator of altered metabolism. This is supported by our gene transcriptional profiling analyses, which show that genes involved in signal transduction pathways are the most significantly changed, followed by those involved in anatomical structural development. The *Pparg* gene, a member of the nuclear receptor peroxisome proliferator activated receptor superfamily, was one of the targets downregulated in our microarray study (Evans et al., 2004; Barish et al., 2006; Ahmadian et al., 2013). Interestingly, although *Pparg* was downregulated, a number of its direct targets, for example, *Mmp13* and *Mmp9*, which are transcriptionally downregulated by *Pparg* (Shen et al., 2012), were upregulated in LBW pups. Pparγ is important for metabolism because it regulates adipogenesis and insulin sensitivity (Barak et al., 1999; Rosen et al., 1999). It has also been demonstrated that Pparγ2 is aberrantly expressed in metabolically relevant tissues of the liver and skeletal muscle when there is altered nutritional exposure (Vidal-Puig et al., 1996; Medina-Gomez et al., 2005). Our analysis reveals a greater extent of Pparγ2 isoform downregulation because it is the regulation of the Pparγ2 isoform that influences nutrition and plays a role in insulin resistance and lipotoxicity (Vidal-Puig et al., 1996, 1997; Ren et al., 2002).

Pparγ forms a heterodimer with Rxr proteins, which upon activation bind PPAR response elements (PPREs) in target genes that regulate lipid metabolism, adipogenesis, insulin sensitivity and induction of *AdipoQ*, a transducer gene that is activated by Pparγ signaling to induce glucose uptake in skeletal muscle. The expression of AdipoQ is decreased when there is insulin resistance (Weyer et al., 2001; Ahmadian et al., 2013). It is noteworthy that AdipoQ was downregulated in LBW pups, and we suggest that downregulation was by direct transcriptional regulation, as ChIP analyses showed a lower level of binding of Pparγ to the promoter of the *AdipoQ* gene.

Epigenetic regulatory mechanisms link the early developmental environment and the growth of the embryo and changes in these regulatory mechanisms increase the risk of developing different diseases in later adult life (Waterland and Michels, 2007; Park et al., 2008; Gluckman et al., 2009). Of all known epigenetic modifications, DNA methylation is the most significant during early fetal development, and Dnmt1 plays a crucial role in the maintenance of methylation (Holliday and Pugh, 1975; Bird, 2002). Our analyses showed that the expression of Dnmt1 was increased both in skeletal muscle and pancreas of LBW pups, and this was confirmed by the observation of the increased level of methylation of the promoter region of the *InsII* gene in LBW pups.

**Fig. 7.**
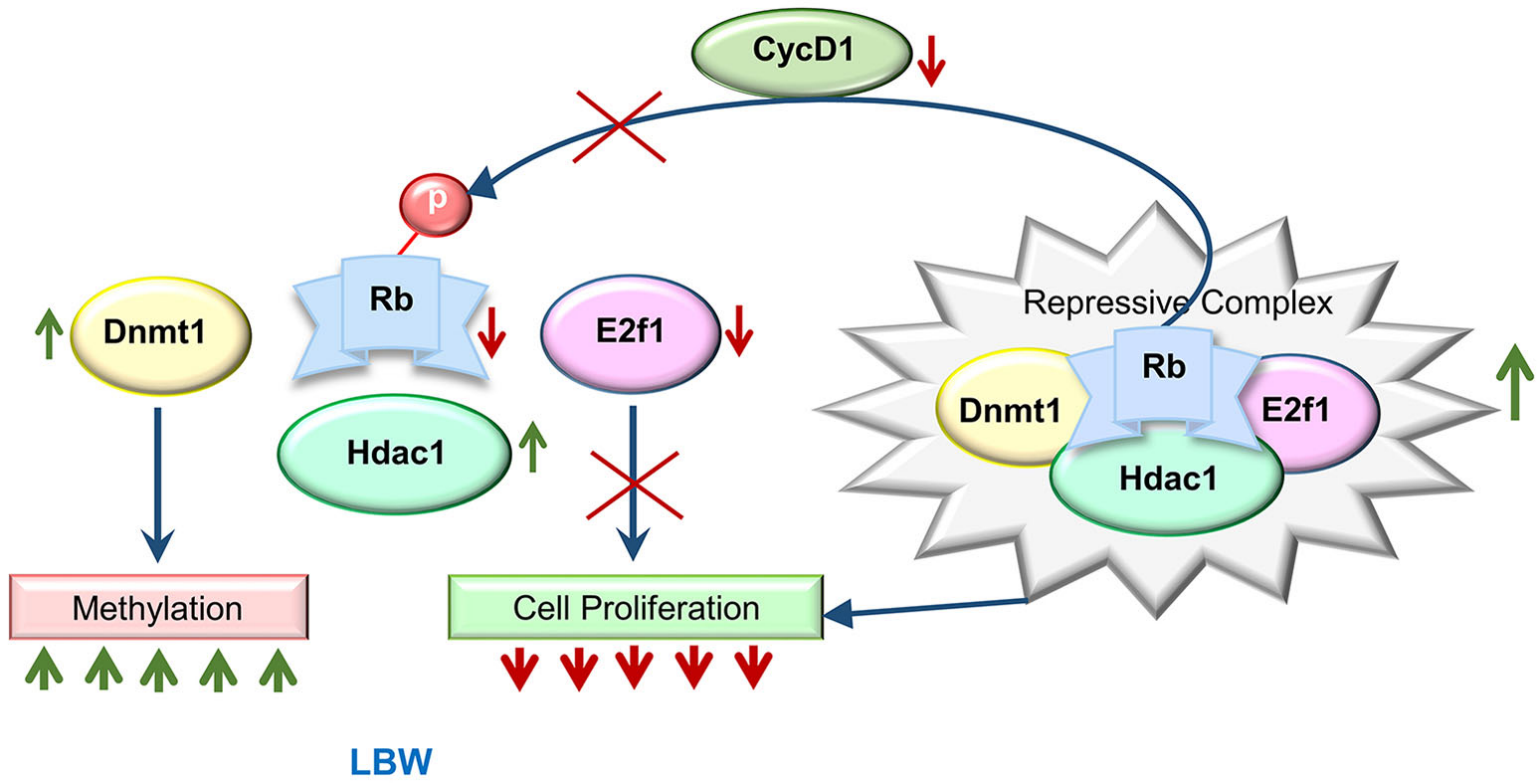
**Possible mechanism by which embryonic growth is coordinated and correlated with the maternal environment.** Elevated Dnmt1 expression might be increasing methylation in the promoters of genes, resulting in suppression of the transcription of genes required for normal growth. In addition, the formation of the Dnmt1 repressive complex with Rb, E2f1 and Hdac1 might slow down cell cycle progression. These processes, probably working together, could be responsible for the growth reduction observed in the birth weight of LBW pups.

An important question of this study was how growth was regulated on a global scale and how birth weight was influenced by the maternal environment. It has been shown that Dnmt1 forms a repressive complex with Rb, E2f1 and Hdac1, which controls the restriction point and therefore progression of the cell cycle (Robertson et al., 2000). Rb protein maintains cell cycle progression from G1 to S phase by regulating the G1 check point or the ‘restriction point’ (Weinberg, 1995). For progress through the restriction check point, E2f1 transcription factor must be made available for initiation of gene transcriptional activity (Brehm et al., 1998; Luo et al., 1998; Magnaghi-Jaulin et al., 1998). The process by which E2f1 is released from the repressive complex for transcriptional activation, leading to progression of the cell cycle (Flemington et al., 1993), requires hyperphosphorylation of Rb protein by cyclin D1/Cdk4 (or Cdk6). Our results demonstrate an increase in the expression of Hdac1 and decreased expression of E2f1 and Rb in LBW skeletal muscle. Further, Co-IP experiments with Dnmt1 showed a greater amount of E2f1 and Rb in the repressive complex in LBW pups. It was also shown that Rb phosphorylation at ser 608 blocks E2F transactivation domain binding (Burke et al., 2010), and ser 780 and ser 795 were phosphorylation targets of cyclin D1-Cdk4/6 (Kitagawa et al., 1996; Connell-Crowley et al., 1997; Garnovskaya et al., 2004), and these phosphorylation levels were low in the LBW pups. Based on these findings, we might conclude that one way by which embryonic growth is coordinated and correlated with the maternal environment is increased Dnmt1 methylation in the promoters of genes, resulting in the suppression of the transcription of genes required for normal growth. In addition, the formation of the repressive complex with Rb, E2f1 and Hdac1 would be expected to slow down the cell cycle and retard embryonic growth. These processes, probably working together, might result in the growth reduction observed in the birth weight of LBW pups (Fig. 7).

## MATERIALS AND METHODS

### General methods

Female (*n*=24, ∼200-210 g) and male (*n*=12, 340 to 350 g) ∼10- to 12-week-old Wistar rats were mated in 12 groups, each consisting of two females and one male. Once pregnant, as determined by the presence of a vaginal plug, female rats were housed in polypropylene cages at 25°C with a controlled light and dark cycle of 12 h, in a purpose-built animal facility of the College of Medicine and Health Sciences, United Arab Emirates (UAE) University.

On delivery, the pups were weighed and grouped as either of lower (LBW) (5th-25th percentile) or average (ABW) (50th-75th percentile) birth weight. Each litter size was adjusted to eight pups per lactating female and maintained with food and water *ad libitum* unless specifically fasted for particular experiments.

For day 1 analyses, two sets of four male LBW and ABW pups were selected. Their birth weights and blood glucose were recorded. The animals were then euthanized by an overdose of anesthetic (pentobarbital or urethane) and their sera, skeletal muscles (the soleus and gastrocnemius muscles taken together in all experiments involving skeletal muscles) and pancreata collected and used for immunohistochemistry, molecular and epigenetic analyses.

Similarly, metabolic parameters were assessed on days 25 (weaning) and 145 (adult), and the tissues were collected after the animals were euthanized as described above.

All animal experiments were reviewed and approved by the Animal Ethics Committee of the UAE University.

### Glucose tolerance test

Three groups, each consisting of four ABW and four LBW 1-day-old pups, were collected very early in the morning and fasted for 6 h. After fasting, blood from the tail vein of one group of four ABW and four LBW pups was assessed for glycemia using a glucometer, after which the pups were euthanized and used as controls. The remaining two groups of LBW and ABW pups were injected with 2 g/kg body weight of glucose intraperitoneally, after which blood from the tail vein was assessed for glycemia at 1 and 2 h after injection, respectively. The pancreata and skeletal muscles were then collected from all the groups for further analysis.

### ELISA

Three groups, each consisting of four ABW and four LBW 1-day-old pups, were collected very early in the morning and fasted for 6 h as above, after which one group was euthanized and the pups’ serum collected as a control. The remaining two groups of LBW and ABW pups were injected with 2 g/kg body weight of glucose and their sera collected. ELISA was carried out using an ultrasensitive rat insulin ELISA kit from Crystal Chem (90060) as per the manufacturer's instructions.

### Isolation of pancreatic islets

Each pancreas was carefully removed and placed immediately in 10 ml washing solution, which was made up of 5% fetal calf serum and 10 mM Hepes in 0.1 M phosphate buffered saline (PBS). The pancreas was cut into smaller pieces and placed in a 15-ml tube containing 2 ml collagenase P solution (1.4 mg/ml, 1129002001, Roche, Indianapolis, IN, USA) on ice. Once the collections were done, they were pooled and incubated for 10 min at 37°C with intermittent vigorous shaking. Approximately 10 ml G solution (HBSS, 0.35 g NaHCO_3_/L and 1% BSA) was then added and the mixture centrifuged for 2 min at 100 ***g*** at 4°C. The pellet arising from the centrifugation was re-suspended in 10 ml G solution and then filtered through a size 40 (420 µm) sieve into a 50-ml conical tube. The total volume was made up to 10 ml with G solution and centrifuged at 130 ***g*** to reform the pellet. The pellet was then re-suspended in 10 ml histopaque 1100 solution (120 ml histopaque 1077 (10771, Sigma-Aldrich, St Louis, MO, USA) and 120 ml histopaque 1119 (11191, Sigma-Aldrich) and centrifuged for 30 min at 210 ***g***. The supernatant containing islets was transferred to a new 50-ml conical tube and centrifuged for 5 min at 300 ***g***. The pelleted islets were washed in 10 ml G solution, centrifuged at 130 ***g*** at 4°C for 10 min and genomic DNA isolated.

### Genomic DNA isolation

Genomic DNA was extracted using a MasterPure™ DNA Purification Kit (MCD85201, Epicentre, Madison, WI, USA) as per the manufacturer's instructions. The isolated pancreatic islet pellet was re-suspended in 300 µl tissue and cell lysis solution in a 1.5-ml micro-centrifuge tube. Then, 1.0 µl proteinase K was added to the sample and incubated for 15 min at 65°C with intermittent vortexing, after which 1.0 µl RNase A was added to the mixture, followed by incubation at 37°C for 30 min. Subsequently, 150 µl protein precipitation reagent was added, mixed and centrifuged for 10 min at 13,680 ***g***. The supernatant was collected and the genomic DNA precipitated with 500 µl isopropanol. The precipitate was rinsed with 70% ethanol and the genomic DNA re-suspended in 30 µl MilliQ water.

### Real-time qRT-PCR analysis

Extraction of total RNA and real-time qRT-PCR was performed as previously described (Emerald et al., 2011). Briefly, total RNA was converted to cDNA with the Applied Biosystem's high-capacity cDNA reverse transcription kit using 1 µg total RNA in a reaction volume of 20 µl as per the manufacturer's instructions (Thermo Fisher Scientific, Waltham, MA, USA). qRT-PCR reactions were carried out in a volume of 20 µl TaqMan^®^ Universal PCR Master Mix with 200 ng cDNA and 200 nM of each primer, using a Quant Studio 7 flex real-time PCR system (Thermo Fisher Scientific). The comparative C(T) method was used to calculate the relative gene expression (Schmittgen and Livak, 2008) and 18S RNA was used as the internal control. The primers used were *18s rRna 4319413E*, *AdipoQ* Rn00595250, *Aadat* Rn00567882, *Bglap* Rn00566386, *Dnaj28* Rn01767146, *Dnmt1* Rn00709664, *Dnmt3a* Rn01027162, *Dnmt3b* Rn015336418, *Edn* Rn00561129, *Gcg* Rn 00562293, *Hdac* Rn 01519308, *Ibsp* Rn00561414, *Ins* Rn 02121433, *Kctd21* Rn01501910, *Lep* Rn00565158, *Mmp13* Rn01448194, *Mmp8* Rn00573646, *Mmp9* Rn00579162, *Ptch1* Rn0141937, *Pparγ* Rn00440945, *Rxra* Rn00441185 and *Ssp1* Rn00681031.

### Western blot analysis

Total protein was extracted with RIPA lysis buffer (1× PBS, 50 mM NaF, 0.5% Na deoxycholate (w/v), 0.1% SDS, 1% IGEPAL, 1.5 mM Na_3_VO_4_, 1 mM PMSF and complete protease inhibitor (000000011836153001, Roche Molecular Biochemicals, IN, USA). Tissue samples were lysed using the RIPA lysis buffer and centrifuged at 9600 ***g*** for 10 min at 4°C. The supernatant was collected and quantitated with a Bio-Rad protein microassay using BSA as a standard (500-0001), and 50 µg of total protein was loaded per lane. Expression of Gapdh was used as a loading control.

Antibodies used were as follows: anti-Dnmt1 (ab92453), anti-Cdk4 (ab131197), anti-Hdac1 (ab7028), anti-cyclin D1 (134175), anti-Glut4 (ab33780), anti-ph-Akt (ab81283), anti-Dnmt3a (ab13888), anti-Dnmt3b ab13604), anti-Gcg (ab92517), anti-ph-IRS1 (ab1194), anti-Akt1/2/3 (ab126811), anti-Pparγ (ab45036) and anti-Rb (ab6075), purchased from Abcam, USA; anti-Pparγ (sc7273) and anti-Rxrα (sc 553), purchased from Santa Cruz Biotechnology, USA; anti-ph-Rb ser780 (RA2133264) and anti-ph-Rb ser 795 (QL2133271), from Thermo Fisher Scientific; anti-Gapdh (2118L), anti-ph-Rb ser 608 (2181s), anti-ph-Akt (4060L), anti-Irs-1 (3407S), anti-Hdac1 (5356s) and anti-P21 (2947s), from Cell Signaling Technology, USA; anti-E2f1 (2651877), from Millipore, USA; anti-AdipoQ (A6354), from Sigma-Aldrich; anti-P16 (10883-1-AP), from Protein Tech, USA; and anti-insulin, from Dako Cytomation, Copenhagen, Denmark. Secondary antibodies used were all purchased from Bio-Rad, USA.

### Membrane fraction isolation

Frozen muscle tissue (50 mg) was homogenized in 2 ml homogenizing buffer [39 ml Buffer A (121.10 mg Tris- base, 37.22 mg EDTA per 100 ml dd H_2_O, at pH 7.4), 13 ml of 20 µM EDTA in buffer A and 312 µl PMSF]. Then, 3 ml buffer 1 (43.5 g KCl, 13.0 g tetra-sodium pyrophosphate in 500 ml dd H_2_O) was added, mixed, set on ice for 15 min and centrifuged at 201,240 ***g*** for 45 min at 4°C. The pellet was washed in 1 ml buffer 2 (121.10 mg Tris-base, 37.22 mg EDTA in 100 ml dd H_2_O at pH 7.4) and the tube was dried with a cotton bud. The pellet was homogenized in 600 µl buffer 2, and then 200 µl 16% SDS was added and centrifuged at 1200 ***g*** for 20 min at 20°C. The supernatant was collected, and its protein concentration determined and used for western blotting.

### Immunohistochemistry

Sections (5-7 µm thick) of pancreata of 1-day-old ABW and LBW rat pups, fixed in Zamboni's fixative were stained by a direct immunofluorescence technique as previously described (Mensah-Brown et al., 2006). Briefly, sections were dehydrated, and after three 5-min washes in 0.1 M PBS, the slides were transferred into 0.1 M citrate buffer and boiled in a 750-W microwave to retrieve antigen for 2×10 min. After cooling at room temperature, the slides were incubated with prediluted insulin and glucagon antibodies overnight at 4°C. The sections were then incubated with secondary antibodies, fluorescein isothiocyanate (FITC)-bound anti-guinea pig IgG and rhodamine (RRX)-conjugated anti-rabbit IgG (Jackson ImmunoResearch Laboratories Inc., USA), both diluted 1:100 in 0.3% Triton X-100 in 0.1 M PBS for 1 h. The specimens were washed three times in 0.1 M PBS for 5 min, mounted and examined by confocal laser scanning microscopy (Nikon C1, Japan). As controls, sections were treated with the universal negative control (N1699, Dako Cytomation, Copenhagen, Denmark).

### Quantification of insulin and glucagon cells

Serial sections (5-7 µm thick) were taken from paraffin wax-embedded pancreata from 1-day-old ABW and LBW pups (*n*=5). Sections (3rd, 6th, 9th, 12th, 18th and 21st) of each pancreata were then immunostained for insulin and glucagon. Islet sizes were determined by measuring their perimeters using an Axiocam digital camera attached to a Zeiss Axiophot.

The total percentages of insulin-containing β cells and glucagon-containing α cells were then determined as follows: area occupied by insulin or glucagon positive cells × 100/area of the whole islet. Every islet in each section examined was included in quantification using the arbitrarily chosen number (*n*=74) of islets in the study.

### Co-IP analysis

For Co-IP analyses, nuclear extracts were prepared from skeletal muscles of day 1 ABW and LBW pups using the universal magnetic Co-IP kit (Active Motif, 54002, Carlsbad, CA, USA) as per the manufacturer's instructions. Antibody binding was carried out with 2 µg of the specific antibody or the control IgG with 800 µg of nuclear extracts overnight at 4°C. Then, 25 µl protein A/G magnetic beads were added, incubated for 1 h, washed and re-suspended in 50 µl of loading buffer, and 20 µl was loaded per lane.

### ChIP analyses

Skeletal muscles from 1 day old ABW and LBW pups were minced and crosslinked for 10 min with 1% formaldehyde in PBS. Crosslinking was stopped by addition of glycine to a final concentration of 0.125 M for 5 min followed by washing with TBS (50 mM Tris pH 7.5, 150 mM NaCl and protease inhibitor cocktail from Thermo Fisher Scientific). After homogenizing the tissue using a Kimble pestle hand-held homogenizer, SDS was added to a final concentration of 0.5%. Chromatin fragmentation was performed by sonication using Bioruptor (Diagenode, Liège, Belgium) for 10 min. Pparγ was immunoprecipitated in Ch-IP dilution buffer (50 mM Tris pH 7.5, 150 mM NaCl, 1 mM EDTA, 1% Triton X-100, protease inhibitor cocktail from Thermo Fisher Scientific) using anti-Pparγ antibody (ab45036, Abcam) or nonspecific IgG control (Upstate Biotechnology, USA) bound to Protein A Dynabeads (Invitrogen, Thermo Fisher Scientific, MA, USA). After 2 h of rotation at 4°C, the beads were washed once with ChIP dilution buffer, once with IP wash buffer (50 mM Tris pH 7.5, 500 mM NaCl, 1 mM EDTA, 1% Triton X-100), once with LiCl wash buffer (50 mM Tris pH 7.5, 250 mM LiCl, 0.5% NP-40, 0.5% sodium deoxycholate, 1 mM EDTA) and finally with TE buffer (10 mM Tris pH 7.5, 1 mM EDTA). Protein-DNA complexes were eluted with 250 µl elution buffer (50 mM Tris pH 7.5, 1 mM EDTA, 1% SDS). Crosslinking was reversed overnight at 65°C and DNA was treated with RNase A and Proteinase K. DNA was purified by phenol/chloroform/isoamyl alcohol and ethanol precipitated. Enrichment was measured by qRT-PCR using SYBR Green PCR core reagent and Quant studio 7 flex real-time PCR system. IP input values were calculated for each sample/primer set and normalized to IP/input values for the negative control primer set (Cha-Molstad et al., 2009). The primers used to amplify the three regions within the *AdipoQ* promoter were: *AdipoQ* R-3Kb 180F 5′-CTGTGTAGCCTGTGGAGCT-3′; *AdipoQ* R-3Kb 180R 5′-CTGGTTAAGACCTTGATTAGTGGG-3′; *AdipoQ* R-3Kb 445F 5′-CTAGTCTAAGGAGAACATGTCGCA-3′;*AdipoQ* R-3Kb 445R 5′-GTCCTATTCTGATTCTCATTGGCT-3′; *AdipoQ* R-3Kb 524F 5′-GCATGAATTGTCTTCGTAAATGAG-3′; *AdipoQ* R-3Kb 524R 5′-ACTGGGAGATCATTTGAAATTTGT-3′; Gapdh ChIP F control primer 5′-ACCATGCTTCACTGACATTCTGA-3′; and Gapdh ChIP R control primer 5′-GGTCTGCCTCCCTGCTAACC-3′.

### Microarray analyses

For microarray analyses, skeletal muscle RNA from 1-day-old ABW and LBW pups (*n*=3) were used. The NimbleGen Rat Gene Expression 12×135K Array (Roche Applied Science, Penzberg, Germany) was used. This array interrogates 26,419 transcripts from Ensembl build RGSC3.4 with five probes per transcript.

The raw microarray data set was normalized and analyzed using in-house R scripts and the Bioconductor package (Gentleman et al., 2004). Genes that were significantly (*P*<0.05) up- or downregulated by a fold change of 1.5 or more were filtered and used to generate heat maps. Over-representation analysis was performed using the Reactome Pathway Database (Croft et al., 2014). The Gene Ontology (GO) Term Mapper (Boyle et al., 2004) tool was used to map GO annotations to higher-level GO slim terms.

### Quantitative methylation profiling of the *InsII* promoter using the Sequenom Epityper System

Methylation of specific CpGs of the *InsII* promoter was analyzed using the Sequenom Epityper System (Agena Bioscience, Hamburg, Germany) according to the manufacturer's protocol. Briefly, 1 µg genomic DNA was bisulphite converted using the EZ DNA Methylation™ Kit (Zymo Research, USA). Bisulphite-treated genomic DNA was PCR amplified with primers tagged with a T7 promoter sequence, transcribed into a RNA transcript and cleaved with a base specific endoribonuclease. During bisulphite treatment, cytosine changes to uracil in the sequence of the unmethylated DNA. This yields a 16-Da mass shift and also generates base specific cleavage products as per the underlying methylation pattern. The cleavage products were then analyzed by matrix-assisted laser desorption ionization time-of-flight mass spectrometry (MALDI-TOF MS). As a measure of reliability of the results, no-template controls, 0% and 100% methylated DNA were included in the assays.

Primer sequences used for the methylation analysis were as follows (Yang et al., 2011): InsII Pro –1136–835F 5′-aggaagagagGGTTTTTTGGTTTTTTTATGGTTTT-3′; InsII Pro –1136–835R 5′-cagtaatacgactcactatagggagaaggctACCTTTCTCAACCTCCACTTAAAAT-3′; InsII Pro –245+118F 5′-aggaagagagATAGTAAAGTTTAGGGGTTAGGGGG-3′; InsII Pro –245+118R 5′-cagtaatacgactcactatagggagaaggctAAAAAACTTCCACCAAATAAAAACC-3′.

### Statistical analyses

All data are expressed as means±s.e.m. of triplicate experiments. Data were analyzed using the two-tailed Student's *t*-test or ANOVA. A calculated *P* value <0.05 was considered significant.

### Accession codes

The complete microarray data have been submitted to the Gene Expression Omnibus (GEO) database under the accession number GSE98851.

## Supplementary Material

Supplementary information
