# Supplementary material for: Identification of early indicators of altered metabolism in normal development using a rodent model system
Source: Dis Model Mech. 2018 Mar 1;11(3):dmm031815. doi: 10.1242/dmm.031815 (PMC5897726; doi:10.1242/dmm.031815)
Supplement: Supplementary information [file dmm-11-031815-s1.pdf]

Table S1 Downregulated genes with a fold change ≥1.5 in LBW pups

No.	NCBI Reference	Gene symbol	Gene name	Expression level	qRT-PCR expression
1	NM_017193	Aadat	Aminoadipate aminotransferase	0.25	0.317
2	XM_001064087	Kctd21	Potassium channel tetramerisation domain containing 21	0.26	0.597
3	NM_001014124	Dnajc28	DnaJ (Hsp40) homolog, subfamily C, member 28	0.28	0.348
4	NM_031010	Alox15	Arachidonate 15-lipoxygenase	0.29	
5	NM_001012029	Ifi204	Interferon activated gene 204	0.32	
6	XM_001061610	Irf4	Interferon regulatory factor 4	0.33	
7	NM_012548	Edn1	Endothelin 1	0.33	0.4197
8	NM_013076	Lep	Leptin	0.34	0.337
9	XM_001078162	Dapp1	Dual adaptor of phosphotyrosine and 3-phosphoinositides	0.34	
10	XM_001056345	Rtp4	Receptor (chemosensory) transporter protein 4	0.34	
11	NM_001008884	RT1-Db1	RT1 class II, locus Db1	0.35	
12	XM_001081280	Ankrd5	Ankyrin repeat domain 5	0.36	
13	XM_001067904	Gpr165	G protein-coupled receptor 165	0.37	
14	NM_139090	Acvr1c	Activin A receptor, type IC	0.37	
15	XM_001076258	Alpk1	Alpha-kinase 1	0.39	
16	NM_001025677	RGD1308782	Similar to Zinc finger protein OZF (POZF-1)	0.39	
17	XM_001064473	LOC683102	Similar to Cd209e antigen	0.39	
18	XM_343566	Tmeff2	Transmembrane protein with EGF-like and two follistatin-like domains 2	0.41	
19	XM_001072594	Gpr81	G protein-coupled receptor 81	0.41	
20	NM_020095	Kcnip2	Kv channel-interacting protein 2	0.41	
21	NM_001015027	Crebl2	CAMP responsive element binding protein-like 2	0.41	
22	NM_053960	Ccr5	Chemokine (C-C motif) receptor 5	0.42	
23	NM_001033961	Kcnip2	Kv channel-interacting protein 2	0.42	
24	NM_182738	Chp2	Calcineurin-like EF hand protein 2	0.43	
25	XM_001056385	Ptchd1	Patched domain containing 1	0.43	0.423
26	NM_013124	Pparg	Peroxisome proliferator-activated receptor gamma	0.43	0.434
27	NM_053391	Hs3st1	Heparan sulfate (glucosamine) 3-O-sulfotransferase 1	0.44	
28	NM_020096	Ifit1	Interferon-induced protein with tetratricopeptide repeats 1	0.44	
29	NM_012689	Esr1	Estrogen receptor 1	0.44	
30	NM_001081451	Fam72a	Family with sequence similarity 72, member A	0.45	
31	NM_001008855	RT1-N3	RT1 class Ib, locus N3	0.45	
32	NM_001005551	Car5b	Carbonic anhydrase 5b, mitochondrial	0.45	
33	XM_001081158	Rad51c	Rad51 homolog c (S. cerevisiae)	0.45	
34	NM_001008858	RT1-T24-1	RT1 class I, locus T24, gene 1	0.45	
35	NM_013108	Adrb3	Adrenoceptor beta 3	0.45	
36	NM_201417	Rxfp1	Relaxin/insulin-like family peptide receptor 1	0.45	
37	NM_022217	Amph	Amphiphysin	0.46	
38	NM_012630	Prlr	Prolactin receptor	0.46	
39	XM_001071139	Layn	Layilin	0.46	
40	NM_001009709	Tmem140	Transmembrane protein 140	0.46	

Disease Models & Mechanisms • Supplementary information

Table S2 Upregulated genes with a fold change ≥1.5 in LBW pups

No.	NCBI Reference	Gene Symbol	Gene name	Expression level	qRT-PCR expression
1	XM_001072242	Mmp13	Matrix metallopeptidase 13	36.97	49.357
2	NM_012881	Spp1	Secreted phosphoprotein 1	32.53	44.150
3	NM_012587	Ibsp	Integrin-binding sialoprotein	26.60	110.778
4	NM_013414	Bglap	Bone gamma-carboxyglutamate (gla) protein	12.14	9.829
5	NM_199398	Panx3	Pannexin 3	9.22	
6	NM_022221	Mmp8	Matrix metallopeptidase 8	8.16	11.448
7	NM_031055	Mmp9	Matrix metallopeptidase 9	7.41	13.434
8	NM_013059	Alpl	Alkaline phosphatase, liver/bone/kidney	6.41	
9	XM_001073586	Tnn	Tenascin N	5.47	
10	XM_001070878	Stfa2	Stefin A2	4.94	
11	XM_001066956	Runx2	Runt-related transcription factor 2	4.80	
12	NM_170668	Slc13a5	Solute carrier family 13 (sodium-dependent citrate transporter), member 5	4.67	
13	NM_053587	S100a9	S100 calcium binding protein A9	4.55	
14	NM_001004129	Stfa2l1	Stefin A2-like 1	4.21	
15	NM_022190	Acan	Aggrecan	4.19	
16	NM_053822	S100a8	S100 calcium binding protein A8	4.14	
17	XM_001075585	Camp	Cathelicidin antimicrobial peptide	3.97	
18	XM_573293	Stfa2l3	Stefin A2-like 3	3.95	
19	NM_019144	Acp5	Acid phosphatase 5, tartrate resistant	3.93	
20	XM_001070786	Stfa2l2	Stefin A2-like 2	3.68	
21	XM_574777	RGD1560095	Similar to double homeobox, 4	3.62	
22	XM_574776	RGD1565550	Similar to double homeobox, 4	3.56	
23	XM_221411	Stfa2l2	Stefin A2-like 2	3.41	
24	XM_001073392	Olfm4	Olfactomedin 4	3.37	
25	NM_057211	Klf9	Kruppel-like factor 9	3.32	
26	NM_001013085	Snx10	Sorting nexin 10	2.96	
27	XM_574775	RGD1560135	Similar to double homeobox, 4	2.91	
28	XM_001075794	Ngp	Neutrophilic granule protein	2.78	
29	NM_181625	Retnlg	Resistin-like gamma	2.76	
30	XM_344827	RGD1563626	Similar to putative protein kinase	2.71	
31	XM_229238	LOC301772	Similar to Y-linked testis-specific protein	2.61	
32	XM_577732	RGD1561339	Similar to putative protein kinase	2.58	
33	XM_344836	RGD1565231	Similar to putative protein kinase	2.53	
34	NM_022528	Hif3a	Hypoxia inducible factor 3, alpha subunit	2.50	
35	XM_001053056	Col10a1	Collagen, type X, alpha 1	2.48	
36	XM_001072193	Anxa9	Annexin A9	2.45	
37	XM_577726	RGD1564848	Similar to putative protein kinase	2.44	
38	NM_013080	Ptprz1	Protein tyrosine phosphatase, receptor-type, Z polypeptide 1	2.44	
39	NM_053968	Mt3	Metallothionein 3	2.44	
40	XM_001065293	LOC685793	Similar to serine/threonine kinase	2.41	

Table S3. List all genes downregulated in LBW pups FC1.5

Click here to Download Table S3

Table S4. List all genes upregulated in LBW pups FC1.5

Click here to Download Table S4

Disease Models & Mechanisms • Supplementary information
